# Immune Cells and Inflammation in Diabetic Nephropathy

**DOI:** 10.1155/2016/1841690

**Published:** 2015-12-28

**Authors:** Zihan Zheng, Feng Zheng

**Affiliations:** ^1^College of Arts and Sciences, University of North Carolina at Chapel Hill, Chapel Hill, NC 27514, USA; ^2^Department of Nephrology, Advanced Institute for Medical Sciences, Second Hospital, Dalian Medical University, Dalian 116023, China; ^3^Department of Nephrology and Basic Science Laboratory, Fujian Medical University, Fuzhou 350002, China

## Abstract

Diabetic nephropathy (DN) is a serious complication of diabetes. At its core, DN is a metabolic disorder which can also manifest itself in terms of local inflammation in the kidneys. Such inflammation can then drive the classical markers of fibrosis and structural remodeling. As a result, resolution of immune-mediated inflammation is critical towards achieving a cure for DN. Many immune cells play a part in DN, including key members of both the innate and adaptive immune systems. While these cells were classically understood to primarily function against pathogen insult, it has also become increasingly clear that they also serve a major role as internal sensors of damage. In fact, damage sensing may serve as the impetus for much of the inflammation that occurs in DN, in a vicious positive feedback cycle. Although direct targeting of these proinflammatory cells may be difficult, new approaches that focus on their metabolic profiles may be able to alleviate DN significantly, especially since dysregulation of the local metabolic environment may well be responsible for triggering inflammation to begin with. In this review, the authors consider the metabolic profile of several relevant immune types and discuss their respective roles.

## 1. Introduction

Diabetic nephropathy (DN) is the most common complication and leading cause of mortality associated with diabetes [1]. DN is a leading contributor to cases of kidney failure in developed countries, and both type I and type II forms of diabetes can result in DN [2]. While the global pattern of DN incidence is not fully surveyed, it is nonetheless a widespread occurrence presently that is expected to increase in prevalence [3]. Numerous factors, both environmental and genetic, have been reported to influence DN onset, severity, and the rate of progression. DN is assessed clinically through a five-stage system of criteria, with each stage featuring a distinct set of functional and structural changes and reflected alterations in benchmark renal function markers [4]. The structural changes begin with glomerular and tubular hypertrophy and the thickening of the basement membrane and mesangium expansion, leading to end-stage glomerular closure and tubulointerstitial fibrosis [5, 6]. These changes are driven primarily by the dysregulation of typical glucose metabolism pathways, leading to the characteristic loss of blood glucose control and aberrant adipose function [7–9]. This loss of glucose control can lead to a number of other changes, including inflammation and cellular stress.

While perhaps not as dramatically observed as in some other conditions, inflammatory processes are heavily involved in the structural deterioration that occurs in DN. This involvement is only natural given that inflammation is known to be involved in the pathogenesis of diabetes, with elevation of serum inflammation markers in long term diabetes patients [10]. The cause(s) of inflammation in DN are not clear, but some combination of pathogen insult and/or tissue damage may be responsible. The former is not likely to be the strongest contributor however, as the kidney is not typically exposed to pathogens and has not been shown to be more vulnerable during DN. Since it is very likely that the inflammation occurring in DN is sterile and chronic, intrinsic kidney cell injury, namely, injury to glomerular, tubular, and vascular cells, may be the main cause. Damage or danger signals released by injured renal cells can trigger remodeling processes by stimulating renal cells and activating immune cells of both the innate and adaptive systems. Other metabolic signals may also contribute. In this review, the authors will review the roles played by, and crosstalk between, several T cells, macrophages, dendritic cells, and renal tubular cells, which are among the most important cell types contributing to the inflammation mediated acceleration of DN progression. The authors will consider the impact glucose metabolism and other mechanisms of metabolic control may have on these different cell types as a key explanatory factor for DN pathogenesis.

## 2. Antigen Presenting Cells

Dendritic cells are the professional APCs in the body, and their function is intimately tied to the progression of diabetic nephropathy. DCs exist primarily in two types: the more common myeloid-derived type (mDC) and the less common, but more highly pathogenic, plasmacytotic (pDC) variety. mDCs and pDCs differ primarily in terms of TNF*α* production, with the high levels of TNF*α* secreted by pDCs having been shown to result in significant morphological changes in DN as well as other autoimmune and inflammatory disorders [11]. More importantly however, DCs can act as keen sensors of damage, being armed with a complete repertoire of receptors for both extrinsic pathogens and intrinsic factors. These include most classes of pattern-recognition receptors, including Toll-like receptors, NOD-like receptors, and C-type lectin receptors, which may also coligate [12]. Following activation by these stimuli, DCs can begin initiating an inflammatory response to counter a perceived threat, potentially via NLRP3 or alternative “licensing” [13, 14]. DC linkage with T cells is sufficient to activate the T cell receptor (TCR) and drive adaptive responses. Signaling along these complex pathways generally ends in the activation of transcription factors such as IRF7, NF-*κ*B, and IRAK, which can then coordinate the secretion of varied chemokines to attract PMNs and alter the behavior of other nearby cells [15, 16]. DCs are able to sense extracellularly through cell surface receptors and intracellularly via endosome-surface receptors. These latter receptors, such as TLR8, may also permit DCs to directly sense microRNAs and other molecules taken up as exosomes [17]. Overall, DC phagocytosis and the ensuing processing mechanisms are critical to the initiation of inflammation. DCs are widely present in the renal tubulointerstitium in normal mice, and immunohistochemistry has also revealed the significant numbers of both mDCs and pDCs in normal human kidneys. As such, resident DCs in kidney may be readily capable of processing damaged cells and signals and consequently initiate the cycle of tissue damage and repair present in DN. DCs have been shown to be critically involved in tubulointerstitial inflammation in various progressive nephritic diseases and in the remnant kidney model. Unfortunately however, the role for DCs in DN inflammation has not been well studied, in part due to difficulties associated with plasticity and the relative instability of cultured DC cell lines [18].

Similar to DCs, macrophages also function as APCs, but few resident macrophages are normally present in the kidneys. Curiously however, a heavily increased macrophage presence is observed in both the glomeruli and tubulointerstitium in human T2D, and the prevention of such macrophage infiltration has been the focus of some recent work [19–21]. Such an increase in the macrophage population has been correlated with the degree of glomerulosclerosis, increased proteinuria, increased serum creatinine levels, and the presence of interstitial fibrosis. Importantly, deletion or inhibition of macrophage accumulation by decreasing intracellular adhesion molecule 1, monocyte chemoattractant protein 1, or colony-stimulating factor receptor 1 ameliorated DN in both db/db and STZ-induced diabetic mice [22]. Given that these knockouts primarily affect chemotaxis and late polarization, it stands to reason that the increase in macrophages around the kidney in DN is primarily a result of outside recruitment, and not simply a local population expansion. Similarly, Smad7 deletion, which impacts the TGF*β* signaling pathway, has been shown to enhance macrophage recruitment to the kidneys [23]. Since outside recruitment typically only occurs in response to damage sensing, it can thus be expected that these macrophages only come into play some time after the initial damage event(s).

Two main types of macrophages, the classically activated and proinflammatory M1 and the alternatively activated and anti-inflammatory M2, are commonly recognized, with the M2 type being further divided into three subtypes. Macrophage populations are generally highly plastic however, such that rigid/exclusive production and expression patterns are rarely observed [24]. Several authors have also suggested various other unique macrophage populations, based on factors such as the differential expression of Lyc6 [25]. Tissue-resident macrophages may also generally express other markers that are tissue-specific, unlike classical bone-marrow derived macrophages [26–28]. Nonetheless, the simple classification scheme is useful for understanding many basic aspects of macrophage function. Transitions along the spectrum from the two extremes have been an area of particular focus and are also highly relevant to the progression of DN. Many of the infiltrating macrophages in the middle stages of DN are likely to adopt the M1 type and cause significant damage that accelerates the condition, although the precise origin of these macrophages is not fully clear.

Many inducers of M1 activity and M1 signature products have been implicated with DN, with TNF*α* being perhaps the most notable. TNF*α* signaling induces production of NF-*κ*B along a JAK/STAT pathway, leading to inflammation. The cytosolic adaptor protein Myd88 conducts the classical pathway for M1 function downstream of TLR signaling. Myd88 may activate various members of the TRAF family, leading to further activation of IRAK as well as IRF7. M1 may also be stimulated via induction of other pattern-recognizing receptors such as NLRs. Regardless of the precise channel used, M1 can be stimulated by both classical pathogen-associated molecular patterns (PAMPs), such as lipopolysaccharide (LPS), and damage-associated molecular patterns (DAMPs) such as extracellular DNA [29]. These stimuli may also elicit different responses in chronic inflammation [30]. The relevance of PAMP-driven signaling to DN mainly lies in possible changes in response to secondary infection, a situation that is not wholly clear. Given the known damage and ECM changes that occur in DN however, it seems that DAMPs are equally important to consider, if not more so.

The transcription factor IRF5 is intimately involved in M1 processes, such that IRF5 is also considered to be one of the defining characteristics of the M1 phenotype [31]. IRF5 is also critical for M1 production of type one interferons and also influences the activity of iNOS, a primary means by which M1 can generate ROS [32]. These signaling molecules work in concert to prevent wound healing but may also prevent hyperresponse via inhibition of other proinflammatory cell types. After all, ROS production has been shown to lead to the inhibition of several T effector subtypes, among others [33]. Despite being proinflammatory, M1 are also sensitive to oxidative stress. M1 are suppressed by an overabundance of NO via the nitration and subsequent loss of function of IRF5 and are actually more abundant in iNOS deficient mice as a consequence [34]. Similarly, hypoxia has been shown to inhibit M1 polarization via altered expression of HIF-1*α* [35]. Oxidative stress has been shown to lead to changes in autophagy and other macrophage activities as well. Interestingly however, high levels of oxidative stress observed in DN have been previously shown to result in tubular cell and podocyte injury, and the debris from cells killed in such manner may also serve to further stimulate M1 and overcome the inhibitory pressure M1 may experience otherwise.

M1 may also produce sizeable amounts of IL-6, but the precise importance of this production is unclear given the fact that many other cell types also secrete the cytokine during inflammation. M1 macrophages can also form inflammasomes to further their function, with the NLRP3 and AIM2 varieties being the ones best characterized thus far. These inflammasomes may form under a wide variety of stimuli and gather together large amounts of caspase 1, which can then cleave and activate interleukins 1beta, 18, and 33 [36]. CARD and PYR domains on these inflammasomes may recruit and involve a broad assortment of different proteins to further M1 function. Damage sensing by M1 macrophages has been demonstrated to contribute to such inflammasome activation, although the exact molecular machinery may be varied and has not been completely characterized [37]. The precise duration of inflammasome activation is also not well understood.

Metabolic control of M1 has become subject of increasing interest and may be particularly relevant to DN given the metabolic disruption that is implicit in the condition. Recent work has shown that M1 macrophages may have an altered expression of the Krebs cycle enzyme isocitrate dehydrogenase, making it a possible target [38]. This altered expression may have important ramifications given the change in glucose availability present in DN. The glucose and salt sensor aldose reductase has also been shown to play a key role in M1 metabolic control, although it is not fully clear if its control is mostly mediated by its production of sorbitol or through other protein interactions [39]. High salt has been demonstrated to result in increased inflammasome activity, but it is not clear if the response is to the salt itself or just to osmolality increase [40]. Advanced glycation end products, commonly observed in diabetes, can also strongly influence M1 macrophage function [41]. It is clear, however, that the provision of high amounts of glucose is a facilitator for M1 stimulation, partly mediated through the increased expression of SREBP1 [42]. These changes may in part be mediated by mitochondrial changes [43]. Interestingly, additional stimulation using insulin may significantly reverse these additions, such that they remain responsive along the standard pathways of metabolism. Arginine metabolism has also been suggested to be involved in M1 control, while other metabolites are probably also relevant to uncertain capacities [44].

In addition, as part of their phagocytotic capacity, macrophages may also uptake large amounts of cholesterol to become foam cells. Foam cells have been identified to occur in a number of renal conditions, and some recent work has focused on promoting cholesterol efflux to prevent foam cell buildup, but it remains to be seen if foam cells should truly be targeted or simply regarded as a marker [45]. In addition, the nuclear receptors FXR, LXR, and PXR have been identified to have important function in regulation of M1, particularly in the Kupffer cells of the liver and in resident macrophage populations in the kidneys. Activation of the bile acid receptor FXR has been shown to repress macrophage function and infiltration capability in DN, partly by suppressing the IFN*γ* mediated activation of STAT1 [46]. However, FXR activation may not be wholly anti-inflammatory, with its mechanistic control still not fully understood. LXR function in macrophages has largely been explored in the context of atherosclerosis, in which LXR activation has been reported to induce efflux of cholesterol from macrophages [47]. LXR activation also influences the activity of the transcription factors IRF8 and Pu.1 [48]. These effects have been confirmed in DN, with LXR activation leading to the amelioration of the condition [49]. This amelioration might be highly dependent on macrophage function, as the AP-1 and NF-*κ*B signaling pathways have been shown to be important in DN, since those pathways feature prominently in macrophages. PXR may also influence macrophage sensitivity to cholesterol, although it may play a more important role in regulating the metabolism of the drugs being used to treat DN given its general role in controlling drug responses [50]. At the same time, activation of these nuclear receptors may also lead to the induction of PPAR family members such as PPAR*α*, which can then serve to negatively regulate inflammation [51]. PPAR*α* has also been shown to influence the self-renewal of erythrocyte progenitors [52]. The balancing of the proinflammatory effects metabolites may have and the anti-inflammatory effects of their activated receptors remains to be clarified.

Overall, macrophages tend to trend towards M1 at the onset of inflammation and then switch to M2 to promote healing some time thereafter. The precise mechanisms that induce such a switch are not understood, although damage sensing, further cytokine stimulation, and autocrine regulation have been proposed as possible reasons [53–55]. Changes in the expression of surface markers such as from CD11b to CD163 may be used to monitor these changes in part but are not wholly sufficient given the persistent expression of perceived M1 markers well after a change in cytokine production has been observed. A large and varied assortment of compounds have attempted to target this transdifferentiation process as a means for alleviating the inflammation in DN and other autoimmune disorders, but none have been extremely successful thus far, despite revealing some interesting target options [56]. Many of these compounds have focused on repressing the activity of M1 transcription factors through selective inhibition, but it is not clear if the transition is actually controlled along a specific pathway. The precise order in which these changes occur is also not fully known.

M2 macrophage differentiation has been one of the key goals for immunology-based treatments for DN, due to the protective effects M2 may induce. In particular, M2 are responsible for the secretion of IL-10, a potent cytokine that can act to suppress the activity of most proinflammatory cell types. IL-10 function runs counter to that of TNF*α*, helping to shut down its efficacy, partly by disrupting the p38/MAPK pathway [57]. IL-10 may also lead to the suppression of iNOS activity via the induction of HO-1 and consequent CO salvage [58]. In addition, IL-10 mediated induction of a PI3K/AKT pathway for cell survival may directly improve wound healing despite otherwise hindering proliferation [59]. Polyamine secretion from M2 may also encourage the resolution of inflammation by influencing transcription programs in other cells [60]. Curiously, M2 function is coordinated to a large extent by IRF4, which is typically understood to be a proliferative and proinflammatory transcription factor across a wide range of different cell types. The exact signal transduction network in play within M2 macrophages that reverses that typical functional pathway is unknown.

## 3. T Helper Cells

T helper cells become relevant at the site of inflammation later than APCs but still have an important functional role in regulating inflammation resolution or lack thereof. T helper cells do not typically have as strong of a residency status as other immune cells, but some tissue-specific markers have been identified. Once activated, CD4+ T cells can organize many of the hallmark signs of inflammation, such as widespread recruitment and chemokine release [61]. Of particular interest in DN is the fact that CD4+ T helpers have been shown to engage in significant crosstalk with fibroblasts to influence fibrosis [62]. As such, targeting against several of these subsets has been shown to be useful in treating DN, particularly Th17 and Th1 [63]. T helper cells can be conventionally split into T effector or T regulatory phenotypes, with the proinflammatory Th1, Th2, Th17, Th9, and Th22 subsets forming the CD4+ subset of the former. Each of these subtypes has the capability of producing signature cytokines, although such production is not necessarily exclusive. Lineage-defining transcription factors may be highly expressed and critically regulate the function of these subtypes, with dynamic interplay between them potentially contributing to the nature of their polarization. The precise strength of TCR signaling at the beginning of their activation has been shown to bias future differentiation towards certain phenotypes [64, 65]. Different metabolites have been demonstrated to also have variable impact across these different phenotypes, although many of those differences remain undiscovered. CD4+ T cells can also be highly plastic, adopting multiple phenotypes either over time or even simultaneously [66]. As such, it is useful to first consider all of the CD4+ cells in context before examining the particular factors that a particular phenotype may hold somewhat exclusively.

CD4+ T cell activation occurs along a multistep pathway, with bona fide activation being somewhat ambiguous. The energy need during this differentiation process, while not well studied, is likely to be quite high, given the clear disparity in metabolic intake between differentiated T effector types and naïve Th0 cells. During this differentiation process, cells may be directly activated and subsequently polarized or potentially undergo a “failed” activation and instead enter into a state of anergy. The nature of CD4+ anergy is not well known, with much of the current information on this phenomenon having been gleaned from analogous study on CD8+ T cells. Anergetic T cells are understood to have very weak, if any, capability of sending further stimulation to other cells, especially via cytokine production [67]. Several types of viral infections are known to induce T cell anergy as a means of escaping immune suppression, and TGF*β* signaling is thought to be involved, but much remains to be clarified. Intriguingly, TLR7 activation following damage sensing has been suggested to induce this state of anergy, implying a potential increase in the population of anergetic T cells in the context of DN [68].

Following activation, T helpers can then pass through a functional stage of variable duration before eventually making a choice to become memory cells or otherwise undergo exhaustion or apoptosis. CD4+ T helper memory is thought to be critically regulated by TSLP, which may induce the cells to adopt a lower metabolic profile [69]. More recently, a newer exhaustion phenomenon has been uncovered, which has been treated as being distinct from simple plastic transitions across different phenotypes [70]. CD4+ T cells are known to require significant autocrine secretion of IL-2 (as well as lesser amounts of IL-5 and IL-7) in order to maintain their viability and continue functioning [71]. In some viral diseases however, T cell function has been observed to fade over time, with IL-2 autocrine signaling being suppressed [72, 73]. This suppression has been correlated with the increased expression of the cell surface marker PD-1 and the transcription factor Bcl6, among others [74]. While some early speculation held that these cells were in fact merely becoming Tfh-like, the observation of other markers such as Tim-3 suggests otherwise [75]. Once CD4+ cells begin to undergo exhaustion, they gradually lose functions one at a time. Recently, several authors have proposed that this type of exhaustion may also occur in cases of chronic inflammation, where the continued activation and function of CD4+ cells may eventually be suppressed through some sort of internal transition. This type of exhaustion-mediated transition has thus far been identified to occur between Th17 and Treg phenotypes but may also occur between other T effector types and T regulatory types [70]. This type of exhaustion is likely caused by some kind of damage sensing mechanism or perhaps the extreme buildup of stress that may occur in chronic inflammation. Deprivation of sufficient nutrients/metabolites may also contribute to exhaustion occurring.

CD4+ T helper tolerance of glucose has been shown to be an important factor in the pathogenesis of diabetes and as such is also highly relevant to DN. In particular, Th17 and Th1 cells have been identified as contributing factors. Th17 cells are a highly active T cell population classically activated through the cytokines IL-6 and TGF-*β* or alternatively activated into a pathogenic form via IL-1*β*, IL-6, and IL-23. As noted previously, these cytokines are often present in significant concentrations in the local environment during inflammation, with significant amounts of them being released by epithelial cells, particularly by those under stress. Once Th17 cells receive signaling along either tract, a JAK/STAT pathway is activated, culminating in STAT3 activation of ROR*γ*t and ensuing production of IL-17 [76]. Changes in the production of IL-17 and in the expression of ROR*γ*t are understood to be the most conclusive indicators of change in Th17 function, but it is also possible that other changes may emerge even earlier. Th17 function has been explored as a possible contributor to worsening DN, but the actual strength of the connection is unclear. Some studies have suggested that Th17 cells might be uniquely upregulated in DN, although it is also possible that the increase is simply a part of a general increase in proinflammatory cytokines [63]. Th1 cells are a similar proinflammatory subset that often responds in a similar fashion to Th17 cells in the face of stimulus. By contrast, Th1 cells produce large amounts of IFN*γ* and express the transcription factor T-bet (Tbx21). Stimulated by the common APC product IL-12p40, Th1 cells rely on STAT4 activation at the end of a JAK/STAT pathway to function. Like other T helper subtypes, Th1 are also influenced by BATF-driven and IRF4-driven transcription networks [77]. Th1 cells have also been shown to be likely candidates for upregulation in DN.

CD4+ T helpers may also respond differently to common forms of oxidative stress common in DN. Th17 cells may also be preferentially activated by the hypoxia environment that can form in DN, although they are also susceptible to inhibition by nitric oxide and other ROS stress [78]. In this manner, they differ from other CD4+ T helpers which are less responsive to NO or otherwise feed off of it to increase inflammation, such as Th9 [79]. Unlike APCs, T helper cells have significantly muted expression of classical DAMP and PAMP receptors, such that they are not as likely to be directly influenced by danger signals such as extracellular DNA. Recent work has shown that human T helpers may be inhibited via activation of TLR7 however, raising hope that other direct influences may also be uncovered. APC transduction of such signaling might occur at a rapid pace in DN however, especially given the broad scale damage that is present during accelerated remodeling. In addition, T cells can directly receive signaling via other extracellular metabolites, such as ATP [80]. Extracellular ATP may then activate the AMPK pathway to increase inflammation by upregulating metabolism [81]. While it is not intuitively obvious that increases in metabolism must correlate with an increase in inflammation, it is almost always the case, as basic increases of metabolism can strongly impact cellular activation [82].

Metabolic control of CD4+ T helpers has been the focus of increased study in recent years, with many metabolic products having been identified to bias cellular polarization. For instance, succinate, an intermediate in the Krebs cycle, has been shown to play an important role in modulating Th17 activity [83]. Provision of excess amino acids has also been shown to somewhat preferentially induce Th17 activation, while its opposite starvation selectively inhibits the phenotype [84, 85]. Other metabolites, such as salt, have also been shown to induce T effector activity, although it is also possible that the change is primarily a result of an increase in osmolality [86, 87]. Glucose can similarly lead to the creation of phenotype bias [88]. Interestingly, in many of these cases, the Th2 and Treg phenotypes are not affected, for reasons not fully clear. One particularly striking example of this phenomenon is the fact that BRD4 inhibition seems to sharply limit Th1 and Th17 activity while leaving Th2 and Treg populations at original levels, despite the fact that BRD4 is broadly expressed across CD4+ lineages and is a very general factor critical for the assembly of the superelongation complex for replication and transcription [89, 90]. It may also be possible that CD4+ T helpers can respond differently to the provision of nucleotides or other metabolites of different classes. Regardless, this differing need for various metabolites leads to a useful potential source for T helper targeting or at the very least cautions against the use of some methods for treatment. For instance, the inhibition of Th17 cells has been a longstanding goal in many conditions besides DN, and one of the prime means for achieving this has been through the treatment of broad-acting corticosteroids. Unfortunately however, recent research suggests that Th17 cells may actually be able to more effectively respond to corticosteroid stress than some other subsets and actually survive such inhibition over the short term [91]. As such, it is important to observe that these metabolic differences can only be targeted in cases where one cell type expresses and requires a metabolite that other polarization states do not also need.

## 4. Tubular Cells

While not immune cells, tubular cells in the kidney may serve to propagate many of the immune signals present in DN, both through direct cytokines and through metabolites. After all, tubular cells handle filtered glucose and other metabolites, while also transporting electrolytes and water. Although currently there are debates about whether or not the primary role of tubular cells is acting in parallel with glomerular cells in the pathogenesis of DN, it is a well received fact that functional dysregulation in the tubules and cell injury are main contributors to DN [92]. As noted previously, one of the early pathologic changes in DN is tubular cell hypertrophy, followed by increased thickness of tubular basement membranes. These changes affect the composition of the extracellular matrix and drive feedback for further changes [93]. Subsequently, tubular atrophy, atubular glomeruli, extensive immune cell infiltration, and fibrosis develop and progress [94]. Multiple factors including hypermetabolism, excessive free radicals, and chronic inflammation have been implicated in DN tubulointerstitial lesions. Naturally, work load in tubular cells in diabetes is greatly elevated as a result of hyperfiltration, along with accumulated high glucose and related metabolites. Accordingly, energy generation and consumption are increased to meet the functional need, leading to a state of hypermetabolism that provokes cell stress and free radical overgeneration [95]. Moreover, nonenzymatic interactions between high glucose pressure and proteins promote the generation of carbonyls in the process, as well as advanced glycation end products which provoke free radical synthesis [96]. This cycle may also cause positive feedback in the absence of a functional response to insulin [97]. Tubular cells may also directly produce TGF*β* in response to glucose to directly modulate immune function [98].

Free radicals are widely recognized to be critical for DN pathology. Free radicals may directly damage cell structures and modify cellular proteins and DNA, resulting in cell stress and injury, which may subsequently activate DCs and macrophages to initiate a pattern of damage associated sterile inflammation [10]. Additionally, free radicals may oxidize nitric oxide (NO) to decrease NO availability and cause vasoconstriction, leading to decreased tubular blood supply and hypoxia. Hypoxia suppresses prolyl hydroxylase domain proteins and activates oxygen sensing transcription factor hypoxia inducible factor (HIF-1*α*). It is noted that free radicals can also increase HIF-1*α* transcription and function by directly activating the HIF-1*α* promoter and regulating hydroxylase function of the asparaginyl hydroxylase [99]. Indeed, the levels of HIF have been noted to increase in the tubular and interstitial cells of DN, and the suppression of HIF has been shown to ameliorate DN [100]. One of important functions of HIF is to switch cell metabolism from oxidative phosphorylation to glycolysis to increase cell survival under hypoxic condition. However, this metabolic shift also causes the changes in immune cell phenotypes that favor inflammation. HIF-family proteins increase macrophage aggregation, invasion, and mobility and are intimately involved in macrophage polarization. For instance, macrophages lacking HIF-2*α* cannot be stimulated via LPS to generate M1 inflammatory responses. The function of HIF in DCs is less clear. Hypoxia induces the production of proinflammatory cytokines TNF*α* and IL-1*β* by DCs. On the other hand, DCs cultured under hypoxia have less costimulatory molecules and maturation markers and show decreased migration in response to C-C chemokine receptor type 7 ligands [101].

Since the process of T cell activation and differentiation is tightly associated with metabolic switch, it is not unexpected to find a role for HIF in T cell activation and differentiation. Th1, Th2, and Th17 cells exhibited increased glycolysis and less oxidative phosphorylation compared to T regulatory cells, which show a greater reliance on lipid oxidation and oxidative phosphorylation [102]. Th17 has high levels of HIF-1*α* and deletion of HIF-1*α* in T cells inhibits Th17 differentiation in vitro and in experimental autoimmune encephalomyelitis [103]. In contrast, hypoxia and deletion of HIF ubiquitination ligase VHL both promote stabilization of HIF-1*α* and enhance generation of Th17 CD4+ cells. The generation of Treg is usually opposite to the induction of Th17 CD4+ cells. Thus, enhanced Treg cell differentiation was observed in the case of HIF-1*α* deletion, a condition not favoring Th17 cells lineage differentiation [104]. One possible molecular mechanism for HIF attenuation of Treg cell development is direct binding and targeting of the Treg lineage-defining transcription factor Foxp3 for degradation [105]. HIF-1*α* can also affect metabolic adaptations in CD8+ T cells and control expression of many molecules associated with effector function and migration [106]. HIF-1*β*-deficient T cells show diminished expression of effector molecules. Overall, HIF activation by hypoxic microenvironment as well as free radicals in tubulointerstitial area favors inflammation via modulation of immune cells.

The levels of HIF are also elevated in tubular cells in DN. It is unknown if HIF also directly promotes the inflammation in tubular cells. However, since the preferred metabolic fuels for tubular cells are not glucose, the act of HIF on these cells may differ. Nevertheless, phenotypic alteration of tubular cells to proinflammatory has been observed in DN. This may contribute directly to local inflammation. Both high glucose and advanced glycation end products can activate NF-*κ*B and inflammasome and upregulate MCP-1 and VCAM-1 in tubular cells, while also promoting further ECM modifications [107]. Large amounts of albuminuria have also been implicated in the proinflammatory phenotype of tubular cells [108].

Finally, apart from genetic susceptibility, DN takes about 10–20 years to develop as the result of multiple vicious interplays among dysregulated metabolism, cell stress and injury, chronic inflammation, and epigenetic changes. Although we have gained some knowledge about the pathogenesis of DN and have developed some treatment in recent years, at present we are still unable to prevent disease progression to diabetic renal failure. As such, it is critical to understand the details of metabolic abnormalities, the pros and cons of HIF activation, and the keys to block chronic inflammation in order to enable more effective treatment of DN.
